# Independent domains of daily mobility in patients with neurological gait disorders

**DOI:** 10.1007/s00415-020-09893-2

**Published:** 2020-06-12

**Authors:** Max Wuehr, A. Huppert, F. Schenkel, J. Decker, K. Jahn, R. Schniepp

**Affiliations:** 1 German Center for Vertigo and Balance Disorders (DSGZ), Ludwig-Maximilians-University, Marchioninistrasse 15, 81377 Munich, Germany; 2 Department of Neurology, Ludwig-Maximilians-University, Munich, Germany; 3 Schoen Clinic, Bad Aibling, Germany

**Keywords:** Daily mobility, Body-worn sensor, Wearable, Factor analysis, Gait disorder

## Abstract

The aim of this study was to establish a comprehensive and yet parsimonious model of daily mobility activity in patients with neurological gait disorders. Patients (*N* = 240) with early-stage neurological (peripheral vestibular, cerebellar, hypokinetic, vascular or functional) gait disorders and healthy controls (*N* = 35) were clinically assessed with standardized scores related to functional mobility, balance confidence, quality of life, cognitive function, and fall history. Subsequently, daily mobility was recorded for 14 days by means of a body-worn inertial sensor (ActivPAL^®^). Fourteen mobility measures derived from ActivPAL recordings were submitted to principle component analysis (PCA). Group differences within each factor obtained from PCA were analyzed and hierarchical regression analysis was performed to identify predictive characteristics from clinical assessment for each factor. PCA yielded five significant orthogonal factors (i.e., mobility domains) accounting for 92.3% of the total variance from inertial-sensor-recordings: ambulatory volume (38.7%), ambulatory pattern (22.3%), postural transitions (13.3%), sedentary volume (10.8%), and sedentary pattern (7.2%). Patients' mobility performance only exhibited reduced scores in the ambulatory volume domain but near-to-normal scores in all remaining domains. Demographic characteristics, clinical scores, and fall history were differentially associated with each domain explaining 19.2–10.2% of their total variance. This study supports a low-dimensional five-domain model for daily mobility behavior in patients with neurological gait disorders that may facilitate monitoring the course of disease or therapeutic intervention effects in ecologically valid and clinically relevant contexts. Further studies are required to explore the determinants that may explain performance differences of patients within each of these domains and to examine the consequences of altered mobility behavior with respect to patients' risk of falling and quality of life.

## Introduction

Disorders of gait and balance and associated mobility impairments are a common complication in neurological diseases and affect around 60% of patients [41]. Highest prevalence for gait and mobility impairments have been reported for patients with Parkinson's disease, followed by vascular encephalopathy, cerebellar ataxia, and sensory deficits. Limitations in mobility function can be debilitating with considerable consequences for patients' functional independence, social participation, and overall quality of life [24, 38]. Moreover, gait impairments in these patients are linked to an increased risk of falling [34, 42] and injuries resulting from falls not only entail substantial medical costs but also determine patients' mortality risk [9].

Disease-specific clinical scales or outcomes from neurological functional diagnostics frequently fail to adequately reflect or predict the degree of mobility restrictions and the risk of falling in different neurological disease cohorts [7, 10, 35]. It appears therefore crucial to directly and comprehensively examine gait and mobility function in these patients. In effect, functional mobility tests (e.g., the Timed Up and Go test [29]) and instrument-based measures of gait stability have been shown to more adequately capture mobility impairments and to more reliably identify a risk of falling in central and peripheral neurological [28, 32, 33, 36, 37, 45] as well as in geriatric patients [1, 13, 25]. However, a drawback of these approaches lies in the fact that they are primarily performed within the laboratory or a specialized clinical setting. They may therefore miss or underestimate the challenges of real-life mobility during which patients actually fall.

In this context, recent advances in daily mobility assessment using body-worn inertial sensors promise a more adequate and specific characterization of patients' mobility impairments in ecologically valid and clinically relevant settings [20, 46]. In contrast to an instrument-based gait assessment in the clinical setting, which focuses on the detailed spatiotemporal features of a patient's gait pattern, body-worn sensors are primarily used to assess patients' mobility from a macroscopic perspective [20]. Accordingly, sensor-based activity monitoring has been focused on measures that allow to quantify how single epochs (also referred to as 'bouts' [3]) of activity (e.g., walking, standing, sitting, lying) alternate and accumulate over time. A plethora of such mobility measures have been proposed in the past (e.g., number of steps, intensity of activity, number and variability of activity bouts, etc. [4, 19, 31, 39]) and there is yet no consensus on what parameters best capture the essence of real-world mobility performance and are clinically most relevant.

Using techniques from factor analysis, Lord and colleagues could previously demonstrate that a large set of available mobility measures can be reduced to a meaningful, small set of independent mobility domains that adequately capture daily-life activity in community-dwelling older adults [19]. Based on this previous work, the aim of the present study was (1) to examine whether an analogous approach might yield a parsimonious and adequate model of daily mobility activity in patients with neurological gait disorders and (2) to explore which explanatory characteristics might determine differences of patients' mobility performance within this model.

## Methods

### Participants

240 patients and 35 healthy controls were recruited as part of a cross-sectional prospective study. Inclusion criteria for patients were the presence of an early-stage, chronic gait disorder caused by either (1) a peripheral vestibular disorder (i.e., chronic or subacute vestibular dysfunction according to the diagnostic criteria [18, 43]; *N* = 66), (2) a cerebellar disorder (i.e., cerebellar ataxia according to the diagnostic criteria [6]; *N* = 72), (3) a hypokinetic disorder (i.e., the diagnosis of idiopathic Parkinson´s diseases, progressive supranuclear palsy or multiple system atrophy according to the respective diagnostic criteria [15, 26]; *N* = 15), (4) a vascular disorder (i.e., white matter hyperintensities with cognitive and postural impairments according to the respective diagnostic criteria [2, 17]; *N* = 49), or (5) a functional disorder (i.e., functional vertigo and dizziness according to the diagnostic criteria [40]; *N* = 38). Further inclusion criteria were the ability to ambulate independently and the absence of any manifest motor weakness of the lower limbs (hemiparesis, paraparesis of the legs). Relatives of patients and employees at the hospital were recruited as healthy controls. All participants gave their informed written consent prior to inclusion.

### Clinical assessment

All participants underwent a standardized interview, which included an inquiry of the following information: ambulatory status, functional status, medication. As part of a retrospective fall risk assessment, information on the number and the severity (based on the Hopkins grading scale [5]) of falls within the past 6 months was collected. Based on this information, participants were categorized with respect to their fall status (non-faller; occasional faller; frequent faller, i.e., N_falls_ $\ge \hspace{0.17em}$2) and with respect to the most severe consequences of falling (no falling = grade 0; near falling = grade 1; falling without requiring medical attention = grade 2; falling requiring medical attention = grade 3; falling requiring admission to the hospital = grade 4).

The subjective level of stability was evaluated by the Falls Efficacy Scale-International (FES-I) and the Activities-specific Balance Confidence scale (ABC-d) [12, 30]. Health-related quality of life was assessed by the Short-Form Health Survey (SF-12) [8]. Cognitive function was screened with the Montreal Cognitive Assessment (MoCA) [27]. Each participant underwent a complete neurological and physical examination including the assessment of functional mobility by the Timed up Go test (TUG) and the Functional Gait Assessment score (FGA) [29, 44].

### Daily mobility assessment

Monitoring of daily mobility was undertaken for 14 days. Participants wore an inertial-sensor-based activity monitor (ActivPAL^®^, PAL Technologies, Glasgow), which recorded the sequence and period of time of individual bouts of ambulatory, sedentary, and sleeping behavior at a sample rate of 10 Hz. The inertial sensor was placed at the thigh of the dominant leg approximately 0.1 m cranial and 0.05 m lateral of the patella. Participants were advised to continue their daily activities as usual and not to change their routine. At the end of the recording period, participants removed the sensor independently and sent it back via postal service.

The following standard parameters (expressed as average daily estimates) were computed from the ActivPAL data in accordance to previously described procedures [19, 31] to represent characteristics of ambulatory, sedentary, and sleeping behavior: intensity, i.e., the amount of energy expenditure expressed as the total metabolic equivalents (METS); step count, i.e., the total number of steps; the number of sit-to-stance transitions (SST); the percentage of ambulatory, sedentary or sleeping time; the number and average duration of ambulatory and sedentary bouts; the distribution of ambulatory and sedentary bouts computed as the Gini Index, which characterizes how total time is accumulated from different bout lengths (a high Gini Index indicates a greater contribution of long bouts to the pattern of accumulation); the within-subject variability of ambulatory and sedentary bout lengths.

### Data analysis

#### Factor analysis

A principle component analysis (PCA) was performed to identify which combinations of activity monitor measures best capture daily-life behavior. PCA is a factorization of the original $m\times n$ data matrix $X$ ($n$ variables; $m$ measurements), such that

$$X = P{W}^{\mathrm{T}} \mathrm{a}\mathrm{n}\mathrm{d} P= XW$$

where $W$ is an orthonormal projection matrix (i.e., ${W}^{T}W=I$) and $P$ is the projection of original $n$-dimensional data matrix $X$ onto the new r-dimensional space defined by $W$. Matrix $W$ is referred to as the *loading matrix* and is computed so that its columns are the directions of maximum variance in the data, with the first column representing the direction of maximum variance, the second column the direction of the next largest variance and so forth. $P$ is referred to as the *factor score matrix*. In total 14 variables (as described above) were included into PCA and varimax rotation was used to derive orthogonal factor scores, with the minimum eigenvalue for extraction set to 1. Items that met a minimum loading of 0.6 were considered relevant [19, 21]. The obtained factor scores were used for further model evaluation.

### Statistical analysis

Descriptive statistics are presented as mean ± SD. Analysis of variance (ANOVA) and Chi-squared tests were used to test for differences of metric and categorial parameters from clinical assessment, daily mobility assessment, and retrospective fall assessment between patients and healthy controls. The obtained daily mobility domains from PCA were compared between patients and healthy controls using ANOVA. Hierarchical regression analysis was performed to identify associations between each mobility domain and explanatory characteristics. Personal characteristics (age, gender, BMI) were entered first, followed by functional scores (FGA, TUG) in the second bock. The third block consisted of falls history (status, grade), and the fourth block included scores for balance confidence, activity level, quality of life, and cognitive function (FES-I, ABC-d, SF-12, MoCA). Standardized beta coefficients and partial correlations were used to evaluate the contribution of each predictor to the variance within each mobility domain. Results were considered significant at *p* < 0.05. Statistical analysis was performed using SPSS (Version 25.0; IBM Corp., Armonk, NY, USA).

## Results

Descriptive statistics on personal characteristics as well as outcomes from clinical assessment and daily mobility monitoring can be found in Table 1. Patients and healthy controls did not differ in basic demographics parameters. In accordance to the recruitment criterion on preserved independent walking ability, patients exhibited only moderately impaired functional mobility scores in clinical assessment. They further yielded near-to-normal cognitive function but reported lower confidence in balance and increased concerns about falling. In correspondence, retrospective fall assessment revealed that patients had considerably more often fallen in the past with significantly more severe consequences of falling (according to the Hopkins grading scale). Parameters from daily mobility monitoring in patients were predominantly within the normal range or only moderately impaired, in particular in terms of a reduced daily duration and intensity of ambulatory behavior.

**Table 1:** Demographic, clinical, and daily mobility characteristics for patients (*N* = 240) and healthy controls (*N* = 35)

Characteristic	Healthy subjects	Patients	ANOVA
Personal characteristics			
Age (years)	52.1 ± 17.7	54.3 ± 15.2	*p* = 0.424
Gender (female/male)	19/16	122/118	
BMI	24.7 ± 3.9	27.0 ± 16.8	*p* = 0.425
Functional mobility scores			
FGA	27.7 ± 4.3	23.7 ± 5.5	***p < 0.001***
TUG	8.7 ± 3.2	10.1 ± 4.9	*p* = 0.099
Fall status			
Status (no/occasional/frequent)	30/3/2	125/46/64	***p = 0.001***
Grade (0/1/2/3/4)	26/4/3/2/0	66/60/69/21/19	***p < 0.001***
Confidence/QoL/cognitive scores			
FES-I	17.6 ± 2.7	27.4 ± 10.4	***p < 0.001***
ABC-d	93.7 ± 11.8	70.2 ± 24.9	***p < 0.001***
SF-12	30.3 ± 3.2	30.9 ± 3.1	*p* = 0.309
MoCA	27.0 ± 2.8	24.8 ± 3.6	***p = 0.001***
Daily mobility measures			
Intensity (METS)	34.5 ± 1.3	33.7 ± 1.6	***p = 0.004***
Step number	9424 ± 3291	7672 ± 3745	***p = 0.009***
SST	40.3 ± 17.7	37.7 ± 15.4	*p* = 0.359
Ambulatory percentage (%)	8.3 ± 2.8	6.8 ± 2.9	***p = 0.006***
Sedentary percentage (%)	28.8 ± 7.4	30.8 ± 9.2	*p* = 0.238
Sleep percentage (%)	39.4 ± 6.7	42.8 ± 9.9	*p* = 0.053
AB number	461.5 ± 148.5	390.4 ± 148.4	***p = 0.009***
SB number	45.3 ± 17.6	41.9 ± 15.4	*p* = 0.242
AB duration (s)	15.9 ± 4.6	15.2 ± 4.0	*p* = 0.296
SB duration (s)	618.2 ± 241.4	712.3 ± 365.2	*p* = 0.132
AB distribution	0.61 ± 0.06	0.60 ± 0.07	*p* = 0.318
SB distribution	0.70 ± 0.05	0.70 ± 0.05	*p* = 0.553
AB variability	1.11 ± 0.09	1.09 ± 0.08	*p* = 0.191
SB variability	1.57 ± 0.11	1.57 ± 0.13	*p* = 0.928

Significant group differences are highlighted in bold font

*BMI* body mass index, *QoL* quality of life, *FES-I* Falls Efficacy Scale-International, *FGA* Functional Gait Assessment score, *TUG* Timed Up and Go test, *ABC-d* Activities-specific Balance Confidence scale (ABC-d), *SF-12* Short-Form Health Survey, *MoCA* Montreal Cognitive Assessment, *AB* ambulatory bout, *SB* sedentary bout, *SST* sit-to-stance transitions

**Fig. 1:**
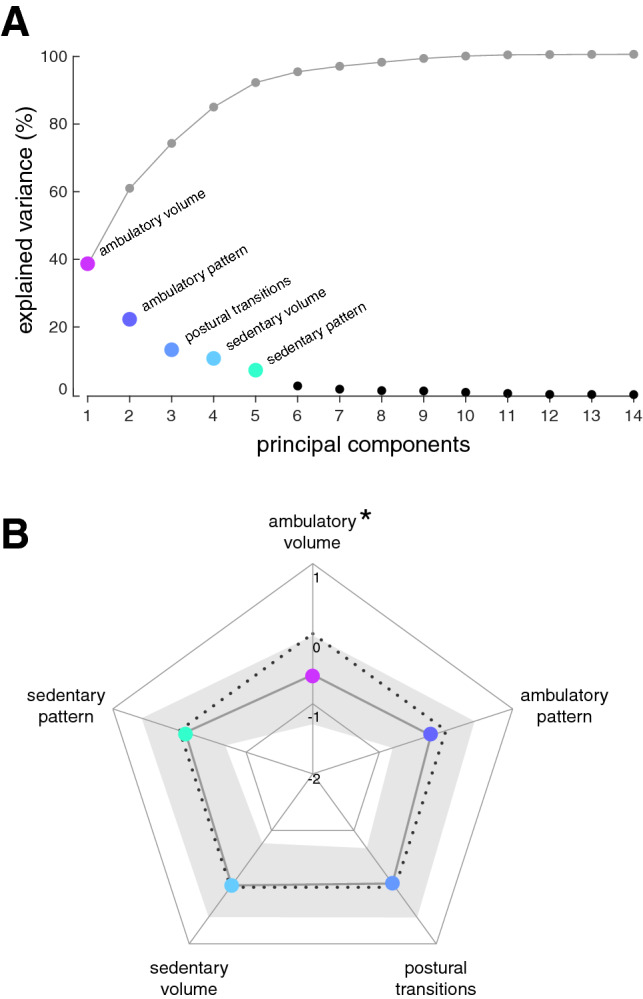
Relative importance of mobility domains and group differences within each domain. **a** Principle component analysis of the dataset with 14 mobility measures from 275 recordings yielded in total 14 components (black dots) with the five first factors (colored dots) explaining 92.3% of total variance (cumulative explained variance is indicated by gray dots). **b** Radar plot with median *z* values (colored dots) and interquartile ranges (gray shaded area) of patients for all five mobility domains. Patient data is normalized with respect to healthy control performance (dotted black line). Daily mobility activity in patients falls within the normal range for all domains except ambulatory volume

Out of the in total 14 included mobility measures, PCA yielded five orthogonal factors that did not exhibit notable cross-loadings. All item loadings were greater than 0.7 (Table 2). The five obtained mobility domains account for 92.3% of the total variance in activity monitor measures (Fig. 1a). We designate these domains as (1) ambulatory volume (i.e., the total amount of ambulatory activity; accounting for 38.7% of total variance), (2) ambulatory pattern (i.e., the temporal distribution and variability of different bouts of ambulatory activity; accounting for 22.3% of total variance), (3) postural transitions (accounting for 13.3% of total variance), (4) sedentary volume (i.e., the total amount of sedentary activity; accounting for 10.8% of total variance), and (5) sedentary pattern (i.e., the temporal distribution and variability of different bouts of sedentary activity; accounting for 7.2% of total variance). Daily mobility performance in patients and healthy controls was compared based on the five resultant daily mobility domains. This analysis revealed that patients only showed reduced scores in the ambulatory volume domain but had near-to-normal scores in all remaining domains.

**Table 2:** Item loadings from principle component analysis for the five mobility domains

Item	Ambulatory volume	Ambulatory pattern	Postural transitions	Sedentary volume	Sedentary pattern
Ambulatory volume					
AB number	**0.908**	− 0.198	0.293	− 0.014	− 0.002
Intensity	**0.885**	0.411	0.172	0.009	− 0.016
Ambulatory percentage	**0.879**	0.382	0.221	0.012	− 0.039
Step count	**0.817**	0.510	0.187	0.007	− 0.026
Ambulatory pattern					
AB duration	0.139	**0.951**	− 0.035	0.027	− 0.059
AB variability	0.202	**0.924**	− 0.007	− 0.041	0.010
AB distribution	0.102	**0.908**	− 0.041	− 0.073	0.058
Postural transitions					
SB number	0.176	− 0.011	**0.937**	0.245	− 0.037
SST number	0.193	− 0.019	**0.927**	0.255	− 0.039
SB duration	− 0.501	0.000	− **0.708**	0.325	− 0.008
Sedentary volume					
Sleep percentage	− 0.357	0.092	− 0.177	**− 0.842**	− 0.040
Sedentary percentage	− 0.459	− 0.003	0.150	**0.839**	− 0.022
Sedentary pattern					
SB distribution	0.080	− 0.044	0.104	− 0.103	**0.947**
SB variability	− 0.230	0.097	− 0.476	0.268	**0.711**

Relevant item loadings are highlighted in bold font

*AB* ambulatory bout, *SB* sedentary bout, *SST* sit-to-stance transitions

In a next step, associations between the obtained mobility domains and various explanatory characteristics were analyzed. For each mobility domain, we found differential moderate associations with demographic items (age, gender), confidence (FES-I), and functional mobility (FGA, TUG) scores (Table 3). These characteristics explained between 19.2% (for ambulatory volume) and 10.2% (for ambulatory pattern) of the total variance within domains. Falls grade (i.e., the most severe consequences of past fall events) and subjective fear of falling (i.e., FES-I) had the overall strongest impact on ambulatory volume. Functional mobility scores (i.e., FGA and TUG) were primarily associated with the two mobility domains related to sedentary behavior.

**Table 3:** Summary of the outcomes of hierarchical regression analysis to identify predictive demographic and clinical characteristics for the five domains of daily mobility performance

Mobility domain	*R*^2^ (*R*^2^ ∆)	Significant predictors	*β*	Partial correlations	ANOVA
Ambulatory volume					
Step 1	0.014	Falls grade	0.315	0.193	*p* < 0.001
Step 2	0.091 (0.077)	FES-I	− 0.311	− 0.180	
Step 3	0.109 (0.018)	Gender	− 0.142	− 0.151	
Step 4	**0.192** (0.083)				
Ambulatory pattern					
Step 1	0.076	Gender	0.170	0.171	*p* = 0.012
Step 2	0.098 (0.022)	Age	− 0.157	− 0.136	
Step 3	0.099 (0.001)				
Step 4	**0.102** (0.003)				
Postural transitions					
Step 1	0.041	Gender	− 0.157	− 0.158	*p* = 0.010
Step 2	0.061 (0.020)				
Step 3	0.086 (0.025)				
Step 4	**0.104** (0.018)				
Sedentary volume					
Step 1	0.076	Age	0.228	0.197	*p* = 0.003
Step 2	0.091 (0.015)	FGA	− 0.222	− 0.142	
Step 3	0.092 (0.000)	Gender	0.159	0.161	
Step 4	**0.118** (0.027)				
Sedentary pattern					
Step 1	0.095	Gender	− 0.273	− 0.271	*p* = 0.002
Step 2	0.103 (0.008)	TUG	− 0.158	− 0.134	
Step 3	0.109 (0.006)				
Step 4	**0.124** (0.015)				

Step 1: age, gender, BMI; step 2: FGA, TUG; step 3: fall status, falls grade; step 4: FES-I, ABC-d, SF-12, MoCA. Resultant model *R*^2^ for each domain is highlighted in bold font

*FES-I* Falls Efficacy Scale-International, *FGA* Functional Gait Assessment score, *TUG* Timed Up and Go test, *ABC-d* Activities-specific Balance Confidence scale (ABC-d), *SF-12* Short-Form Health Survey, *MoCA* Montreal Cognitive Assessment

## Discussion

Assessment of gait and mobility function is gaining increasing importance for clinical diagnostics or monitoring of disease progression, as a measure for the efficacy of interventions, and as a marker for identifying those patients at a high risk of falling. However, for gait as well as for daily mobility assessment, clinicians are often unable to decide, which of the plenty available outcome measures are the most appropriate for each of the above-mentioned applications [20]. In the past, factor analysis techniques have been successfully applied to conceptualize both types of assessment, in terms of a low-dimensional set of independent functional domains (typically 3–5) that adequately cover the essential aspects of the general gait pattern at an microscopic and the everyday mobility performance at an macroscopic perspective [14, 19, 21, 45]. However, most of these conceptualizations and models were developed based on datasets derived from the healthy elderly population, which yet impedes a direct application to the clinical field of neurological gait and balance disorders.

Here we demonstrate that the approach of low-dimensional modelling of real-world mobility behavior can be extended to patients with different forms of neurological gait disorders. Our obtained model includes five independent functional mobility domains, which we designate as ambulatory volume, ambulatory pattern, postural transitions, sedentary volume, and sedentary pattern. This model shares several communalities to the previously presented model from Lord and colleagues that was obtained from a dataset on community-dwelling older adults [19]. In particular, in both models, measures of ambulatory and sedentary activities as well as measures of transitions between these activities group in separate functional domains. Intuitively, quantities of ambulatory and sedentary behavior should yield redundant information as they apparently represent opposing ends of the very same phenomenon. However, both models emphasize that the characterization of sedentary behavior and postural transitions between activities entails complementary information that is required to comprehensively capture the pattern of activities during daily-life routine. In contrast, to Lord's model that is composed of only three domains (i.e., ambulatory behavior, sedentary behavior, and postural transitions), the current model comprises five functional domains inasmuch as ambulatory and sedentary activities are each further differentiated into a domain that reflects the volume and intensity of behavior and a domain that represents the distribution and regularity of behavior. This differentiation is in accordance with previous reports that indicate that both the volume and the diversity of activities reflect different aspects of the general mobility status of individuals [11, 22, 31]. Thus, the discrepancy between the two models presumably results from the apparently different ambulatory status of the two study populations. Accordingly, reduced activity levels in the elderly population in Lord's study might eventuate in generally fewer degrees of freedom within the pattern of daily activities and a collapse of otherwise functionally independent domains of behavior [16].

The current analysis focused on a patient cohort with an early-stage manifestation of different neurological gait impairments with yet retained independent ambulatory status (predominantly from a population at working age). This selection criterion was reflected in the obtained model, according to which patients exhibited only moderately reduced scores in the ambulatory volume domain compared to age-matched healthy participants but near-to-normal scores in all remaining domains. Thus, patients in our cohort tended to maintain their everyday routine (i.e., a near-to-normal pattern of different activities over the day) despite showing initial signs of a disease-related reduction on mobility volume. Regression analysis revealed that reduced performance in ambulatory volume domain was associated to a lower balance confidence and increased concerns about eventual falls. This association indicates that not only fall-related injuries that actually impair mobility function but already a lower balance confidence resulting from an incipient decline of postural function and/or from former falls may considerably affect the amount of everyday mobility [19]. Moreover, higher scores of ambulatory volumes in patients were associated to more severe consequences of falling. In accordance with previous reports, this finding suggests that in particular for patients with early-stage gait disorders a longer exposure to walking situations entails a higher risk for the occurrence of frequent and especially of severe fall events [23].

Consistent with previous work, the overall outcome of regression analysis on mobility domains was only modest [19]. Despite including a comprehensive set of demographic, subjective confidence, and clinical characteristics, we did not identify explanatory characteristics that would convincingly account for individual performance differences along the dimensions of the five different mobility domains. With respect to functional mobility scores (i.e., FGA and TUG), this observation indicates that real-world measures of mobility provide complementary information on patients' mobility status that is not readily available from clinical evaluation. Counterintuitively, patients' quality of life as assessed by the SF-12 score did not at all show any association to the different aspects of patients' daily-life activity. This observation might at first glance contradict the high expectations on long-term mobility assessment as an specific and patient-relevant outcome measure for future clinical trials [46]. However, our focus on a clinical population with predominantly early-stage gait disorders that exhibited near-to-normal daily mobility performance and quality of life scores certainly limits this observation. It is indeed conceivable that more pronounced impairments of daily mobility in advanced stages of neurological gait disorders may be actually more strongly associated to alterations in patients' quality of life. Further studies that include a more comprehensive study population and more specific measures of health-related quality of life are therefore required to corroborate or disprove this observation.

In conclusion, the here established model for daily mobility behavior in neurological gait disorders may provide a convenient framework for future studies on disease-specific mobility impairments and their consequences for patients' well-being, quality of life, and risk of falling. The mainly negative results from regression analysis demand for further research to explore the characteristics that may sufficiently explain performance differences within the proposed functional mobility domains.
